# TP-CSO: A Triptolide Prodrug for Pancreatic Cancer Treatment

**DOI:** 10.3390/molecules27123686

**Published:** 2022-06-08

**Authors:** Xinlong Wang, Huahui Zeng, Xin Zhu, Duanjie Xu, Qikang Tian, Can Wang, Lingzhou Zhao, Junwei Zhao, Mingsan Miao, Xiangxiang Wu

**Affiliations:** 1 Academy of Chinese Medicine Sciences, Henan University of Chinese Medicine, Zhengzhou 450046, China; wangxl_115@126.com (X.W.); hhzeng@hactcm.edu.cn (H.Z.); 2 Pharmacy College, Henan University of Chinese Medicine, Zhengzhou 450046, China; xerz2@126.com (X.Z.); xuduanjie123@163.com (D.X.); hucmkang@163.com (Q.T.); wangjiucan@163.com (C.W.); 3 Department of Nuclear Medicine, Shanghai General Hospital, Shanghai Jiao Tong University School of Medicine, Shanghai 200080, China; zlz-330@163.com; 4 Department of Clinical Laboratory, The First Affiliated Hospital of Zhengzhou University, Zhengzhou 450052, China; junweizhao@alumni.sjtu.edu.cn

**Keywords:** triptolide, toxicity, water solubility, pharmacokinetics, pancreatic cancer

## Abstract

Triptolide (TP) is a potential drug candidate for the treatment of cancer, but its use was hampered by its systemic toxicity and poor water solubility. Hence, a TP-CSO prodrug was synthesized by conjugating TP to chitosan oligosaccharide (CSO), and characterized by ^1^H NMR, FTIR, DSC and XRD analyses. The TP-CSO containing about 4 wt% of TP exhibited excellent water solubility (15 mg/mL) compared to TP (0.017 mg/mL). Compared with TP, the pharmacokinetics of the conjugate after oral administration showed a three-fold increase in the half-life in the blood circulation and a 3.2-fold increase in AUC _(0–∞)_. The orally administered TP-CSO could more effectively inhibit tumor progression but with much lower systemic toxicity compared with TP, indicating significant potential for further clinical trials. In conclusion, CSO-based conjugate systems may be useful as a platform for the oral delivery of other sparingly soluble drugs.

## 1. Introduction

Pancreatic cancer (PC) is a malignant tumor of the digestive tract. The morbidity and mortality of PC rank among the top of malignant tumors [1,2,3]. Currently, GNP (gemcitabine with albumin-bound paclitaxel) and FOLFIRINOX (5-fluorouracil, leucovorin, irinotecan, and oxaliplatin) has been widely used as first-line chemotherapy regimens for the treatment of advanced pancreatic cancer [4,5]. However, pancreatic cancer can evade cell death due to its potential drug resistance, so the clinical effectiveness of this treatment isn’t satisfactory [6,7]. Therefore, it is of great significance to develop a drug that can reduce drug resistance to pancreatic cancer and improve its tumor suppressive effect.

Triptolide (TP) is one of the main bioactive ingredients of *Tripterygium wilfordii Hook F* (*TWHF*) in the Euonymus family. Recently, TP has attracted immense attention to its anticancer activity with multiple mechanisms, which can reduce drug resistance for cancers [8,9,10,11]. Several studies have shown that TP is more effective against pancreatic cancer compared with gemcitabine, paclitaxel, cisplatin and doxorubicin [12,13,14]. However, its clinical development has been greatly hampered by its poor water solubility (0.017 mg/mL) [15], multi-organ toxicity [16,17,18] and low bioavailability.

Recently, a polysaccharide has been developed adequately as a carrier for the clinical application of hydrophobic drugs. The chemotherapy drug-conjugated chitosan, such as LMWC-DTX, LMWC-PTX, exhibited superior antitumor efficacy and less toxicity compared with the parent paclitaxel (docetaxel) [19,20]. Chitosan is a biocompatible, biodegradable, and low-toxic natural polysaccharide. Drug delivery vehicles based on various derivatives of chitosan are currently being explored. Recently, we reported on an oral triptolide-conjugated carboxymethyl chitosan prodrug (CCTP), in which CC acted as a carrier [21]. The CCTP conjugate showed better water solubility (5 mg/mL), lower toxicity, but slightly weaker anticancer effect than the free TP. Chitosan oligosaccharide (CSO, MW: about 3000 Da), low molecular weight chitosan, exhibits nontoxicity, better water-solubility, and a high rate of assimilation in the human gastrointestinal tract [22]. Thus, chitosan oligosaccharide was used as a drug carrier to develop a triptolide-conjugated CSO prodrug (TP-CSO). In our work, we hypothesized that TP-CSO might display several advantages for oral administration, including by dramatically enhancing the water solubility of TP, the significantly reduced toxicity of TP, the prolonged retention of the drug in the blood, and the improved bioavailability and antitumor efficacy in vivo.

Usually, the TP-CSO conjugate may differ from its parent TP in terms of its pharmaceutic efficacy and physicochemical properties. Here we describe the synthesis, characterization, and in vitro drug release profiles of TP-CSO conjugates with preliminary evaluation in mice, aiming to develop a technological platform for the oral delivery of TP.

## 2. Results and Discussion

### 2.1. Preparation and Characterization of TP-CSO

**Scheme 1:**
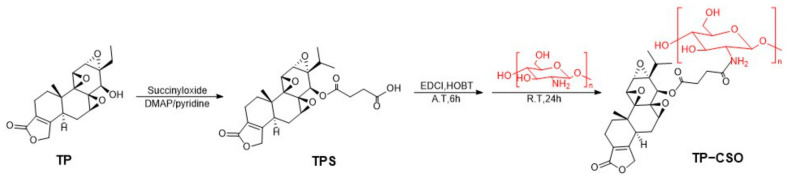
Schematic illustration of the synthetic pathway of TPS and TP-CSO conjugate.

**Figure 1:**
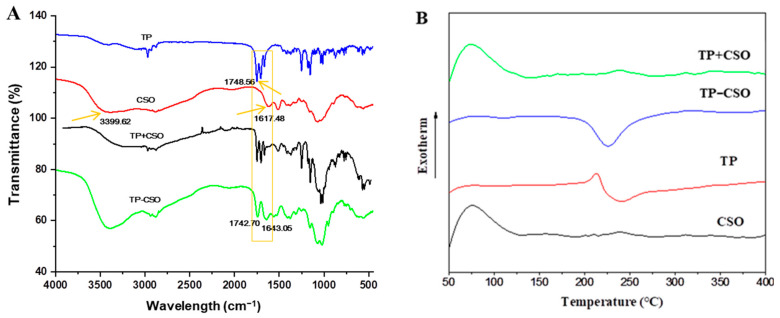

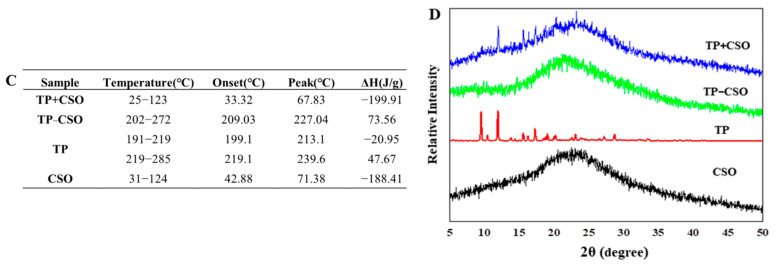
FTIR spectra of TP, CSO, TP+CSO, TP-CSO (**A**); DSC thermogram patterns of TP, CSO, TP+CSO, TP-CSO (**B**); DSC data of TP, CSO, TP+CSO, and TP-CSO (**C**); XRD diffraction patterns of TP, CSO, TP+CSO, and TP-CSO (**D**).

As shown in Scheme 1, TP-CSO was prepared by a coupling reaction between TPS and CSO, and TP could be released via hydrolysis. The structure information of TP, CSO, TP+CSO (physical mixture) and TP-CSO was shown in Figure 1 (^1^H NMR see Supplementary Materials, Figure S1). TP shows a strong absorption band at 1748.56 cm^−1^ that was associated with the C=O stretching of cyclic ester groups. The characteristic peaks of the CSO at 3399.62 cm^−1^ was due to the stretching vibration of NH_2_ and OH groups. Moreover, the 1617.48 cm^−1^ absorption band was corresponded to the N-H deformation of amino groups. The FTIR spectra of TP-CSO showed a new absorption peak at the wavenumbers of 1643.05 cm^−1^, which is attributed to the C=O stretching vibration of the amide group, while the absorption band at the wavenumber of 1617.48 cm^−1^ (corresponding to the NH_2_ group of CSO) mostly disappeared in the FTIR spectrum of TP-CSO, which indicated that the amide group was successfully introduced (Figure 1A).

The thermal behaviors of TP, CSO, TP-CSO and physical mixture (TP+CSO) were investigated using DSC under a nitrogen atmosphere (Figure 1B). The exothermic peaks of the CSO and physical mixture (containing 4 wt% of TP and 96 wt% of CSO) were in the range of 67–71 °C. However, the free TP showed a sharp exothermic peak at 213.1 °C and a broad endothermic peak at 239.6 °C, respectively. The TP-CSO conjugate displayed a broad endothermic peak at 227.04 °C with an onset at 209.03 °C (Figure 1C). Thus, the endotherm of TP-CSO reflected the physical and chemical structure changes during the introduction of the TP moiety into the CSO backbone. In addition, the physical mixture and CSO had the enthalpy (∆H) of -188.41 J/g, whereas TP-CSO had ∆H of 73.56 J/g (Figure 1C). This result could be explained by the fact that increasing acylation caused an increase in enthalpy, which was consistent with the previous report [23]. The XRD patterns of TP, CSO, TP-CSO and the physical mixture (TP+CSO) were depicted in Figure 1D. TP showed many small XRD peaks between the 2theta range of 9.54–28.69°, whereas CSO exhibited two continuous peaks at 21.47° and 24.56°. Compared with TP and CSO, the physical mixture (containing 4 wt% of TP and 96 wt% of CSO) displayed the multiple XRD peaks from the overlap between TP and CSO. Therefore, the broad single XRD patterns of the TP-CSO reflected the decrease in crystallinity of the TP-CSO backbone, which could be attributed to the presence of TP moiety. The XRD patterns revealed that TP-CSO were amorphous compared to TP and CSO. Furthermore, the result indicated that the TP moiety was successfully introduced into the CSO backbone.

The weight percentage of TP in TP-CSO was measured at approximately 4 wt% by using the HPLC method (for HPLC spectra see Supplementary Materials, Figures S2 and S3). The TP-CSO conjugate, a yellowish solid, has over a 35-fold greater water solubility (15 mg TP-CSO/mL) than TP (0.017 mg/mL). The above results showed that the TP-CSO conjugate had been successfully synthesized through the coupling of TP and CSO, which significantly improved the water solubility of TP.

**Figure 2:**
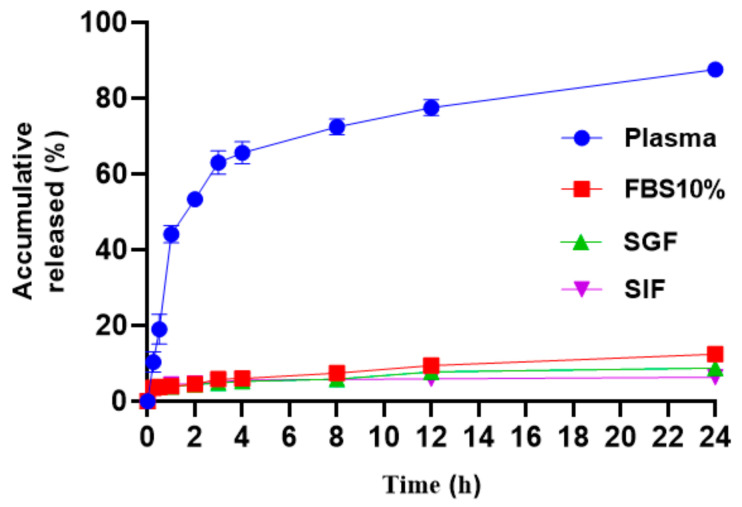
TP-CSO release profiles in mouse plasma, 10% FBS of cell culture medium, SGF (simulated gastric fluid, pH 1.2), and SIF (simulated intestinal fluid, pH 7.5) (n = 3).

In order to investigate the in vitro release behavior of TP-CSO, we measured the drug release of TP-CSO in mouse plasma, cell culture medium, SGF, and SIF at 37 °C (Figure 2). The TP released from TP-CSO was detected by the HPLC in different solutions, which had the same retention time as the native TP, showing that the ester bonds between TP and CSO were cleaved by enzymatic attack. A small amount of TP released from the TP-CSO conjugate was observed after 24 h post incubation in cell culture medium (12.45%), SGF (8.81%) and SIF (6.41%), while 87.58% of TP was observed after 24 h incubation in mouse plasma. The results showed that TP-CSO was quite stable in the cell culture media SGF and SIF, but was easily hydrolyzed in mouse plasma. Owing to the low drug release in SGF and SIF, TP release in SGF and SIF would seem insubstantial, indicating that most of the TP release did not take place in the gastric or intestinal environment, but might mainly transport to the blood capillary.

### 2.2. Pharmacokinetics after Gavage Administration of TP-CSO to Rats

**Figure 3:**
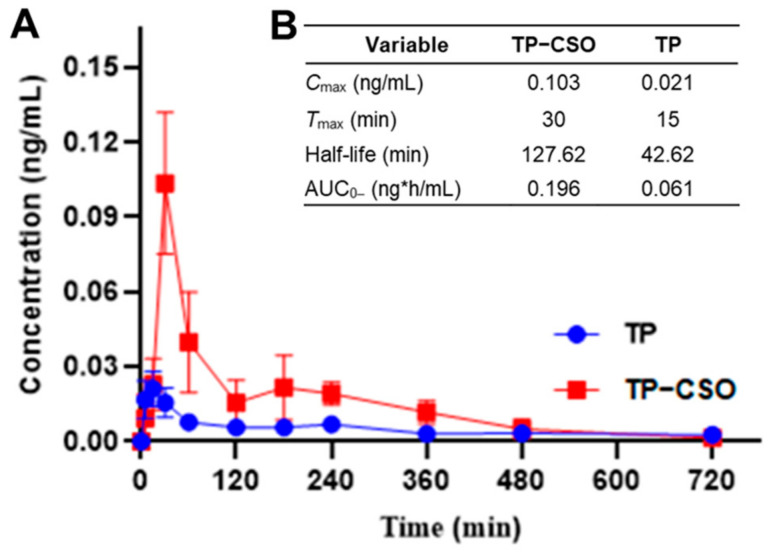
Plasma concentration of TP (**A**), pharmacokinetic parameters (**B**) after administration of TP and TP-CSO, respectively (n = 5).

The pharmacokinetic parameters after gavage administration of TP and TP-CSO in rats were investigated by the LC-MS method to quantify the plasma level of the free TP. As shown in Figure 3A, two concentration curves of TP exhibited a rapid increase, followed by a slow decrease. Meanwhile, the TP-CSO group showed higher detectable concentrations compared with the TP group over a long period of time, suggesting that TP-CSO has higher gastrointestinal absorption and sustained TP release. The relevant pharmacokinetic parameters (e.g., T_max_, C_max_, t_1/2_ and AUC_0–∞_) are shown in Figure 3B. The peak time (T_max_) of TP was half shorter than that of TP-CSO, whereas the peak concentration (C_max_) of the conjugate was five-fold higher than that of TP. In addition, the apparent half-life of TP-CSO (t_1/2_ = 127.62 min) was three times longer in the plasma than that of TP (t_1/2_ = 42.62 min), indicating TP-CSO showed a slow clearance of TP from the blood. The AUC_0–∞_ of TP-CSO (0.196 ng·h/mL) was over three times higher than that of TP (0.061 ng·h/mL), suggesting a high absorption of TP-CSO, which may be attributed to the increase of its water solubility and the high absorption rate of CSO in the gastrointestinal tract.

### 2.3. In Vivo Toxicity of TP-CSO

**Figure 4:**
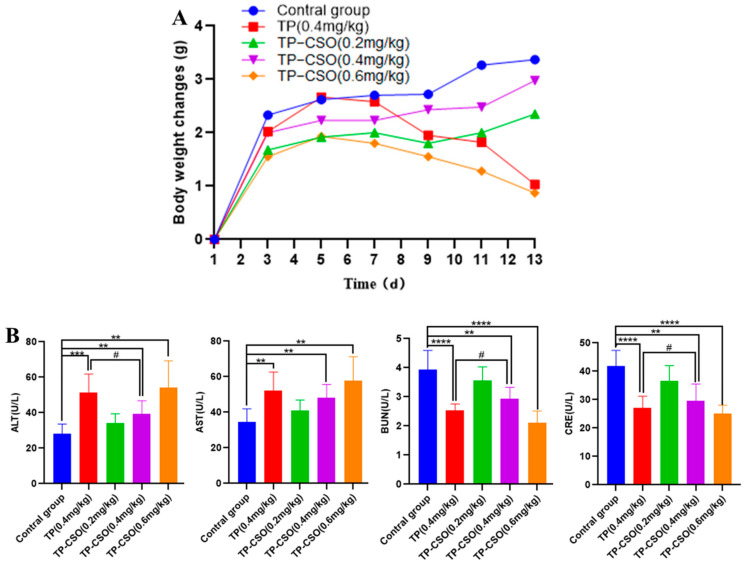
In vivo toxicity of TP and TP-CSO: body weight changes (**A**); biochemical parameters (**B**). ** Represents *p* < 0.01, *** represents *p* < 0.001, **** represents *p* < 0.0001, vs. control group; ^#^ represents *p* < 0.0001, TP-CSO (0.4 mg/kg) vs. TP group (0.4 mg/kg) (n = 6).

The in vivo toxicity of TP and TP-CSO was evaluated in healthy C57 mice (Figure 4A). According to our preliminary experiments, the minimum dose of TP causing the death of mice was about 0.6 mg/kg. There was no significant difference in body weight between the low- and medium-dose TP-CSO groups and the control group (*p* < 0.05), while the TP group and the high-dose TP-CSO group showed obvious body weight loss but without lethality at the end of the experiment, indicating that TP-CSO protected mice from weight loss or death caused by TP-induced toxicity.

Different degrees of liver and kidney injury are indicated by the changes in serum ALT, AST, BUN and CRE levels [24]. The ALT and AST levels in the TP group and the high-dose TP-CSO group were obviously higher than those in other groups, which indicated the two groups occurred liver injury (*p* < 0.05, Figure 4B). However, the low- and medium-dose of TP-CSO groups showed moderate effects on ALT and AST in comparison to the control group (*p* < 0.05), which were still safe for the liver. In the TP group and high-dose TP-CSO group, BUN and CRE levels were significantly lower than in the control group (Figure 4B). The low-dose TP-CSO group had a slight effect on BUN and CRE, whereas the medium-dose TP-CSO group showed higher BUN and CRE levels in comparison to the TP group. The study results showed that in vivo toxicity of TP is related to hepatic and renal injury in a dose-dependent manner. As compared with the TP group, the medium-dose TP-CSO group underwent mild changes in AST, ALT, BUN and CRE levels, indicating that TP-CSO may relieve the TP-induced toxicity in liver and kidney.

### 2.4. Antitumor Activity of TP-CSO

**Figure 5:**
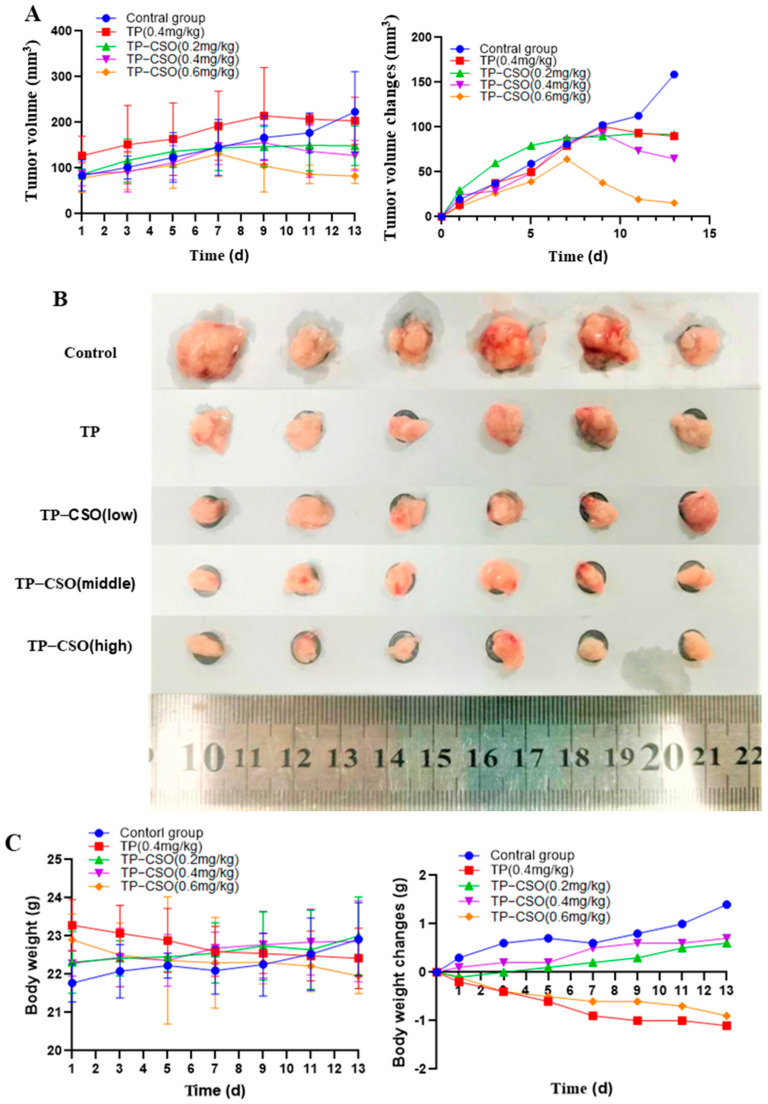

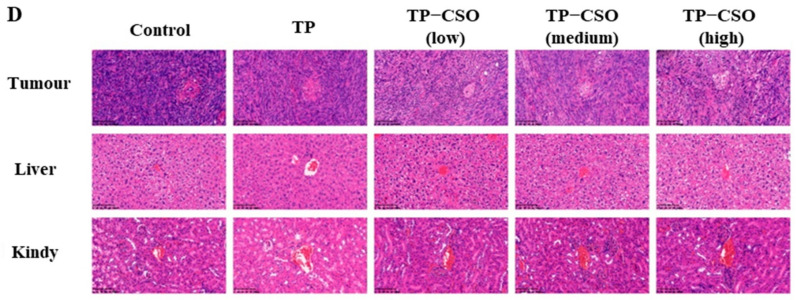
Changes in tumor volume of different drug-treated groups for 14 days (**A**) and on the last day (**B**); mouse body weight change with time (**C**); H&E staining of liver and kidney organs and tumors (**D**).

The in vivo antitumor efficacy of TP-CSO was assessed by monitoring changes of tumor volume in the Panco2-bearing mice (Figure 5). The tumor volume gradually increased in all treatment groups for the first seven to nine days, followed by a decrease in tumor volume (Figure 5A). Compared to the control group, the tumor in all treated groups was significantly diminished in the dose-dependent manner (*p* < 0.05), especially in the high-dose TP-CSO group (Figure 5B). By the end of the study, the tumor-inhibition rate (TIR) was 43.39% in the TP group, 42.18%, 59.23%, and 90.29% in the low-, medium-, and high-dose TP-CSO groups, respectively. At the same dose of TP, TP-CSO had a stronger tumor inhibition effect than the free TP, indicating that much more TP-CSO conjugate were actively or passively accumulated in the tumor cells, followed by TP release from the conjugate via cleavage of the succinate linker between TP and CSO.

In general, body weight in treated groups changes due to a combination of tumor development and drug toxicity. The control group displayed a gradual increase in body weight over 13 days, which showed that the tumor development didn’t primarily cause mainly weight loss (Figure 5C). There was no significant difference between the TP-CSO groups (0.2 and 0.4 mg/kg) and the control group (*p* < 0.05), whereas the TP group (0.4 mg/kg) and the TP-CSO group (0.6 mg/kg) showed an obvious decrease in body weight, indicating the result of the dual effects of both the TP toxicity and its dose dependency. The results suggested that TP-CSO had a better anti-tumor effect and lower in vivo toxicity than TP. We hypothesized that the controlled release of TP-CSO would reduce the instantaneous TP concentration in the blood and prolong the TP circulation time in the blood, which could improve its biological safety and antitumor activity.

As shown in Figure 5D, the control group showed the obvious nuclear division, and negligible apoptosis levels were observed in the dense tumor cells, whereas the treated groups exhibited a large number of cell necrosis and apoptosis in the tumor tissues, indicating TP and TP-CSO have a significant inhibitory effect on tumor cells. In particular, the medium- and high-dose TP-CSO groups displayed a higher apoptosis rate and cell necrosis than the TP group, indicating that TP-CSO has better anti-tumor activity than TP. Analysis of histological staining revealed the obvious kidney proximal tubular dilation and fatty degeneration in the hepatocytes in the TP group (Figure 5D). However, no significant histopathological changes were observed in the TP-CSO groups, which was consistent with the in vivo toxicity results of TP-CSO.

## 3. Materials and Methods

### 3.1. Materials

Triptolide was provided by Xi’an Haoxuan Biotechnology Co. Ltd. in Xi’an, China. Chitosan oligosaccharide (CSO, MW: about 3000 Da) was purchased from Aladdin (Beijing, China) and purified using dialysis. Hyclone fetal bovine serum, Trypsin, Dulbecco’s Modified Eagle’s Medium (DMEM), and Phosphate Buffered Saline (PBS 1X, sterile) were all obtained from Beijing Solarbio Science and Technology Co. Ltd. (Beijing, China). Dimethyl sulfoxide, acetonitrile and methanol were MS-grade reagents provided by Tedia. The water used in the experiments was prepared by a Milli-Q ultrapure water instrument (Millipore, Bedford, MA, USA). Hepatocytes (HL7702) and mouse pancreatic cancer cells (Panco2) were purchased from American type cultures (Manassas, VA, USA). Other chemicals were of commercially available analytical grade.

### 3.2. Preparation and Characterization of TP-CSO Conjugate

A 2′-hemicsuccinic acid derivative of TP (TPS) was prepared according to our previously reported method [19]. In short, 360 mg of TP (1 mmol), 400 mg of succinic anhydride (4 mmol), and 24 mg of 4-dimethyla- minopyridine (0.2 mmol) were dissolved in 10 mL of pyridine. The mixture was stirred for 24 h under nitrogen purging at room temperature. After the reaction was completed, the solvent was removed, and the residue was purified by silica gel column chromatography.

TPS: ^1^H NMR (500 MHz, DMSO-d6) δ 12.23 (s, 1H), 4.99–4.95 (m, 1H), 4.89–4.81 (m, 1H), 4.78 (dd, J = 17.7, 3.5 Hz, 1H), 3.95 (d, J = 3.2 Hz, 1H), 3.71–3.66 (m, 1H), 3.56 (d, J = 5.6 Hz, 1H), 2.65–2.53 (m, 2H), 2.52–2.43 (m, 3H), 2.22 (dt, J = 15.1, 5.9 Hz, 1H), 2.16–2.07 (m, 1H), 2.00–1.92 (m, 1H), 1.87 (p, J = 6.9 Hz, 1H), 1.80 (dd, J = 15.1, 13.3 Hz, 1H), 1.36–1.22 (m, 2H), 0.91 (s, 3H), 0.86 (d, J = 6.9 Hz, 3H), 0.74 (d, J = 6.9 Hz, 3H).

CSO (300 mg, 0.1 mmol) was dissolved in 8 mL of DMSO at 55 °C and cooled down to room temperature after complete dissolution [22]. TPS (100 mg, 0.22 mmol) dissolved in 8 mL of anhydrous DMSO was activated by EDCI (90 mg, 0.46 mmol; 1-ethyl-3-(3-dimethylamino-propyl) carbodiimide hydrochloride) and HOBt (120 mg, 0.88 mmol; N-Hydroxy benzotriazole) for 6 h at room temperature. Subsequently, the activated TP-SUC solution was added into the CSO solution. The mixture was reacted at room temperature for 24 h. The reaction mixture was then dialyzed three times against ultrapure water in a dialysis bag (molecular weight cut-off: 500 Da). The TP-CSO conjugate was lyophilized into a yellowish solid, followed by a rinse with acetone (3×). The dried products were characterized using the 1H NMR and FTIR. The weight percent of TP in TP-CSO was obtained by high-performance liquid chromatography (HPLC) at 218 nm.

TP-CSO: ^1^H NMR (500 MHz, D_2_O) δ 7.32 (s, 1H), 5.34 (d, J = 3.6 Hz, 1H), 4.83 (s, 5H), 4.48 (s, 1H), 4.09 (s, 1H), 3.92 (s, 1H), 3.85 (d, J = 12.1 Hz, 8H), 3.75 (d, J = 9.8 Hz, 1H), 3.68 (q, J = 12.5, 9.9 Hz, 7H), 3.61 (s, 1H), 3.59 (s, 3H), 3.40 (dd, J = 19.3, 7.2 Hz, 4H), 3.22 (dd, J = 10.5, 3.3 Hz, 1H), 3.14–2.97 (m, 3H), 2.91 (d, J = 8.1 Hz, 7H), 2.78 (s, 3H), 2.62 (s, 66H), 2.39 (s, 2H), 2.21 (d, J = 8.3 Hz, 1H), 1.97 (s, 5H), 1.95 (d, J = 4.0 Hz, 1H), 1.89 (d, J = 15.0 Hz, 1H), 1.83–1.77 (m, 1H), 1.46 (s, 1H), 1.28 (s, 2H), 0.98 (t, J = 7.2 Hz, 2H), 0.89 (s, 9H), 0.83 (d, J = 6.9 Hz, 1H), 0.71 (s, 5H).

### 3.3. Differential Scanning Calorimetry (DSC)

DSC curves for TP, CSO, TP-CSO and the physical mixture (TP+CSO) were obtained using a DSC 823e instrument (Mettler-Toledo 823e, Greenville, SC, USA). The powder samples of 20 mg were placed under a stream of nitrogen gas and heated from 50 °C to 400 °C at a heating rate of 5 °C/min prior to the analysis.

### 3.4. X-ray Diffraction (XRD)

Crystalline characteristics of the TP, CSO, TP-CSO and physical mixture (TP+CSO) were investigated by powder X-ray diffraction (D2 PHASER, Bruker Corporation, Karlsruhe, Germany) by a lamp at λ = 1.54 Å. The scan speed was set at 5 s and at 0.02 of increment. The diffraction pattern was set in the range of 5–50° (2θ) at 25 °C.

### 3.5. Determination of TP Loading Capacity

10 mg of TP-CSO was dissolved in 3 mL deionized water and then shocked for 5 min, ultrasonically dissolved for 5 min, and centrifuged at 4000 r/min for 3 min. The supernatant of 300μL was diluted with 2.7 mL redistilled water and measured by HPLC. Relations between physical quantities are given in the form of equations, as follows:

(1) $$w_{TP}=k*A_{TP}$$

(2) $$\mathrm{TP}\;\mathrm{Loading}\left(\%\right)=\frac{W_{TP}}{W_{TP-CSO}}\times 100\%$$

the peak area of TP was represented with *A_TP_*. The linear relation between the peak area and concentration of TP was represented with *k*. The weight of TP-CSO and TP were represented with *W_TP-CSO_* and *W_TP_*, respectively. The drug loading capacity was calculated according to Equation (2).

### 3.6. The Water Solubility Study

For the water solubility studies, TP-CSO (30 mg) was dissolved in 1.0 mL ultrapure water. The mixture was treated using ultrasonic waves for 15 min, vortexed for 3 min, and centrifuged at 12,000 rpm for 10 min. Saturated supernatants were quantified by UV-vis spectrophotometer at 218 nm excited wavelength.

### 3.7. In Vitro Release Study of TP-CSO

To investigate the release of TP-CSO, we assessed the stability of TP-SCO under various conditions. Briefly, TP-CSO (8 mg) was dissolved in 2.0 mL of the mouse plasma, cell culture medium (Containing 10% FBS) and simulated gastric juice (SGF; 0.32% pepsin, pH 1.2) as well as simulated intestinal fluid (SIF; 1% trypsin, pH 7.5), respectively. 100 μL samples were taken after 0.5, 1, 2, 4, 8, 12 and 24 h of incubation at 37 °C, and extraction of 200 μL of dichloromethane. HPLC was performed on an Agilent C-18 column (4.6 × 250 mm, 5 μm) with acetonitrile/water (33:67, 1 mL/min) as mobile phase.

### 3.8. Pharmacokinetic Study

To evaluate the in vivo pharmacokinetics of TP-CSO conjugate, the SD rats (250–300 g, Sprague-Dawley rat) fasted 12 h in advance and were randomized into two groups (n = 5) and respectively treated with TP (0.4 mg/kg) and TP-CSO (0.4 mg/kg, TP equivalent). The blood samples (500 µL) were collected through the ocular chorionic vein at 5, 15, 30, 60, 120, 180, 240, 360, 480 and 720 min after intragastric administration. Subsequently, the samples were immediately centrifuged at 3500 rpm for 15 min. An upper plasma sample was collected and kept at −80 °C.

Sample processing: to the plasma sample,10 µL of ferulic acid (2.5 ng/mL) was added as internal standard. The sample (100 µL) was placed in a 1.5 mL Eppendorf tube, to which 800 µL of ethyl acetate was added and vortexed for 3 min, and this was centrifuged at 12,000 rpm for 15 min. The upper solution was taken and blow dried with nitrogen at room temperature. The dried residue was dissolved in 100 µL of methanol-water (60:40, *v*/*v*) solution, vortexed for 3 min, subsequently centrifuged at 12,000 rpm for 15 min at 4 °C and the upper solution was collected into a 2 mL vial.

10 μL of sample was injected into a Watets C-18 column (2.1 mm × 50 mm, 1.8 µm) on a Sciex X500R QTOF Mass Spectrometer (UPLC-MS; Framingham, MA, USA). The column was eluted with methanol (A)/water (B) solution at a flow rate of 0.3 mL/min and a column temperature of 35 °C (0–6 min, A: 40%–90%; 6–8.1 min, A: 90%; 8.1–10 min, A: 40%). The operating conditions of UPLC-MS are as follows: electrospray ionization source (ESI), negative ionization mode; Ion spray voltage (ISV), 3500 V; Drying temperature, 325 °C; Flow rate of desolvent gas, 8.0 L/min; Atomization temperature, 350 °C. The pyrolysis energy and impact energy of TP were 120 V and 10 V, respectively, and those of ferulic acid were 90 V and 10 V, respectively. A multiple reaction monitoring mode (MRM) was used to monitor the transformation of TP from m/z [M-H] 359.1→241.1 and ferulic acid m/z [M-H] 193.1→134.1 to productions.

### 3.9. In Vivo Toxicity Study

The 30 normal C57 mice (20–25 g) were randomly divided into a normal saline group, TP (0.4 mg/kg) group and TP-CSO group (equivalent TP of 0.2, 0.4 and 0.6 mg/kg), respectively. The drugs were administered by a gavage once every two days for seven times. The body weight and fatality rate were recorded daily. On the 14th day, blood was collected from the eyes, and the organs were harvested after the mice were euthanized. A Beckman CX5 automated analyzer detected renal function indexes, liver function indexes (serum alanine aminotransferase (ALT), aspartic aminotransferase (AST)), and (blood urea nitrogen (BUN) creatinine (CRE)).

### 3.10. In Vivo Antitumor Study

The antitumor activity of the TP-CSO conjugate was evaluated on Panco2 tumor-bearing mice. First, C57 mice (20–25 g) were implanted subcutaneously with 2 × 10^6^ Panco2 cells. When the tumor grew up to about 100 mm^3^, the mice were orally administrated with 0.4 mg/kg TP and TP-CSO (0.2, 0.4 and 0.6 mg TP equivalent/kg) once every two days, respectively. The body weight and fatality rate were recorded. Mice in control groups were orally administrated with saline solution. The tumor volume was measured with calipers and calculated by the (1/2) (a × b²) formula (a is long diameter, b is short diameter). After 14 days of intragastric administration, the mice were sacrificed, and livers, kidney, and tumors were harvested.

### 3.11. Statistical Analysis

All the data in this study were presented as mean and standard deviation. Differences among groups of data were compared by student’s *t*-test or analysis of variance. A value of *p* < 0.05 was considered significant.

## 4. Conclusions

A TP-CSO prodrug was synthesized and characterized by ^1^H NMR, FTIR spectrum, DSC and XRD analysis. TP-CSO exhibited over a 35-fold water solubility (15 mg/mL) in comparison with TP. The bioavailability of TP-CSO after oral administration was higher than the parent TP along with the prolonged circulation time in the blood. Consequently, compared with the same dose of TP, TP-CSO has lower toxicity and a more potent tumor-suppressive effect, showing significant potential for further clinical trials. In conclusion, the CSO-based conjugate system may be useful as a platform for oral delivery of other sparingly soluble drugs.

## Data Availability

The data presented in this study are available upon request from the corresponding author.

## Supplementary Materials

The following supporting information can be downloaded at: https://www.mdpi.com/article/10.3390/molecules27123686/s1, Figure S1: ^1^H NMR spectra of TPS, CSO and TP-CSO; Figure S2: Standard curve for TP; Figure S3: HPLC spectra of TP weight percentage in TP-CSO (sig = 218, 4).
