# Supplementary material for: TP-CSO: A Triptolide Prodrug for Pancreatic Cancer Treatment
Source: Molecules. 2022 Jun 8;27(12):3686. doi: 10.3390/molecules27123686 (PMC9227231; doi:10.3390/molecules27123686)
Supplement: Supplementary file 1 [file molecules-27-03686-s001.zip › molecules-1754757-supplementary.pdf]

Supplementary data

TP-CSO: a Triptolide Prodrug for Pancreatic Cancer Treatment

Xinlong Wang[1,2], Huahui Zeng[2], Xin Zhu[1], Duanjie Xu[1], Qikang Tian[1], Can Wang[1], Lingzhou Zhao[3], Junwei Zhao[4], Mingsan Miao[1,2,*] and Xiangxiang Wu[1,2,*]

1 Pharmacy College, Henan University of Chinese Medicine, Zhengzhou 450046, China

2 Academy of Chinese Medicine Sciences, Henan University of Chinese Medicine, Zhengzhou 450046, China

3 Department of Nuclear Medicine, Shanghai General Hospital, Shanghai Jiao Tong University School of Medicine, Shanghai 200080, China

4 Department of Clinical Laboratory, The First Affiliated Hospital of Zhengzhou University, Zhengzhou 450052, Henan, China

* Correspondence: wuxx-415@126.com; miaomingsan@163.com; Tel.: +86-0371-65680206

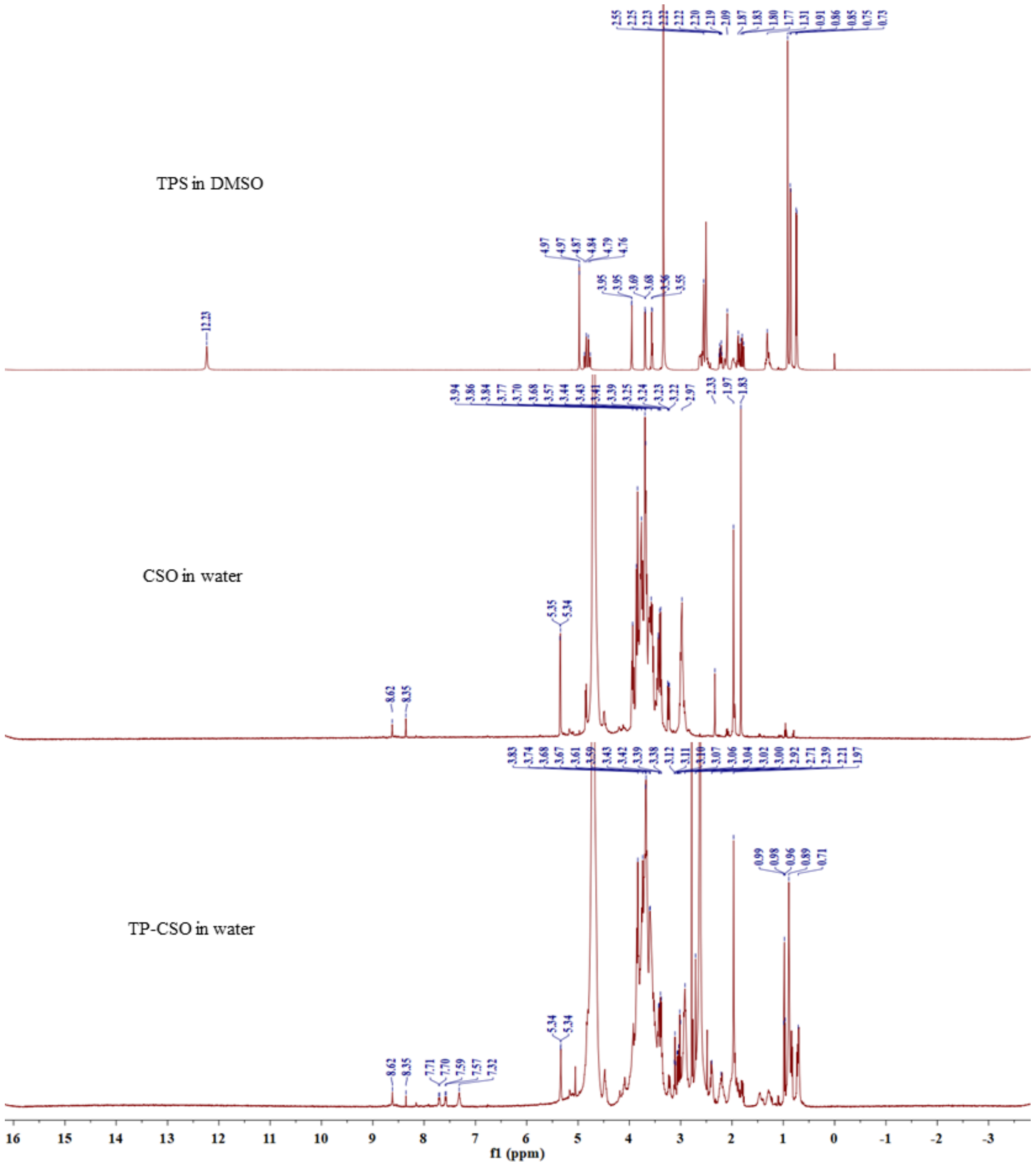

Figure S1: ^{1}H NMR spectra of TPS, CSO and TP-CSO

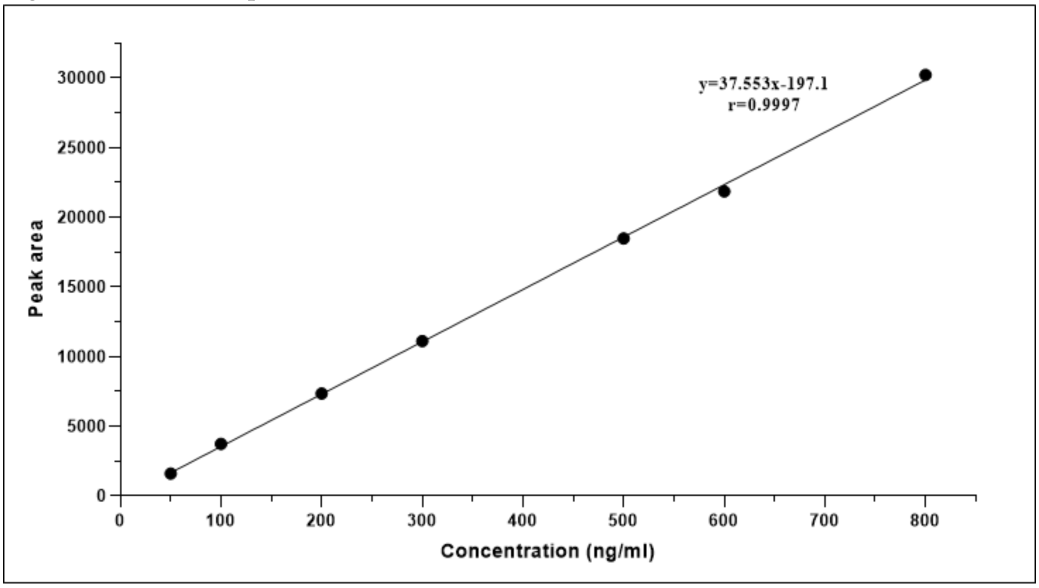

Figure S2: Standard curve for TP

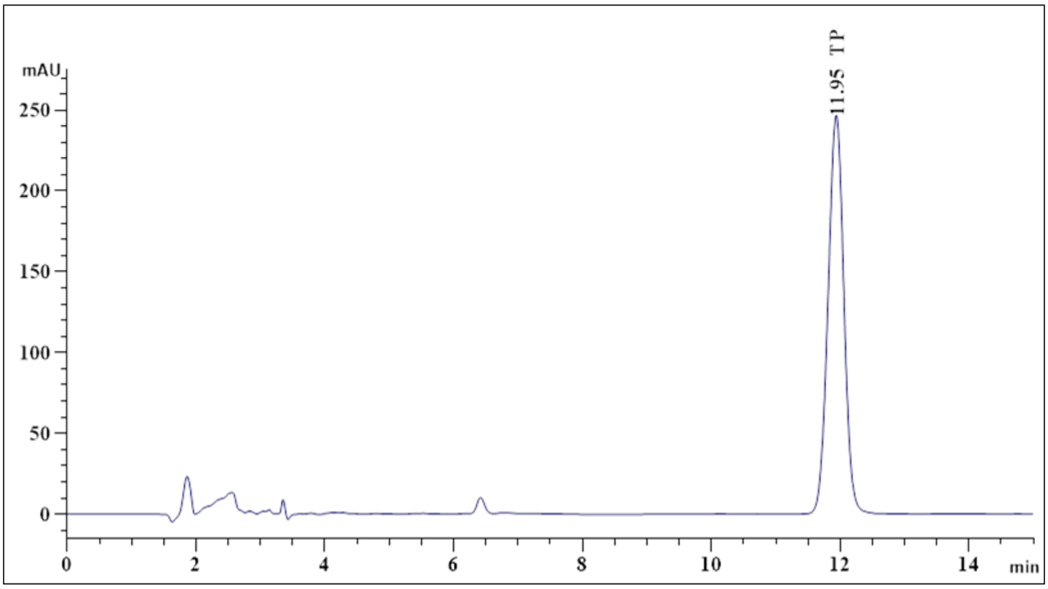

Figure S3: HPLC spectra of TP weight percentage in TP-CSO (sig=218, 4)
